# Oncolytic Activities of Host Defense Peptides

**DOI:** 10.3390/ijms12118027

**Published:** 2011-11-16

**Authors:** Sammy Al-Benna, Yechiel Shai, Frank Jacobsen, Lars Steinstraesser

**Affiliations:** 1 Laboratory for Molecular Oncology and Wound Healing, Department of Plastic Surgery, BG University Hospital Bergmannsheil, Ruhr-University Bochum, Bochum 44789, Germany; E-Mails: sammy.al-benna@ruhr-uni-bochum.de (S.A.-B.); frank.jacobsen@ruhr-uni-bochum.de (F.J.); 2 Department of Biological Chemistry, The Weizmann Institute of Science, Rehovot 76100, Israel; E-Mail: yechiel.shai@weizmann.ac.il

**Keywords:** molecularly targeted therapies, carcinoma, sarcoma, tumor

## Abstract

Cancer continues to be a leading source of morbidity and mortality worldwide in spite of progress in oncolytic therapies. In addition, the incidence of cancers affecting the breast, kidney, prostate and skin among others continue to rise. Chemotherapeutic drugs are widely used in cancer treatment but have the serious drawback of nonspecific toxicity because these agents target any rapidly dividing cell without discriminating between healthy and malignant cells. In addition, many neoplasms eventually become resistant to conventional chemotherapy due to selection for multidrug-resistant variants. The limitations associated with existing chemotherapeutic drugs have stimulated the search for new oncolytic therapies. Host defense peptides (HDPs) may represent a novel family of oncolytic agents that can avoid the shortcomings of conventional chemotherapy because they exhibit selective cytotoxicity against a broad spectrum of malignant human cells, including multi-drug-resistant neoplastic cells. Oncolytic activity by HDPs is usually via necrosis due to cell membrane lysis, but some HDPs can trigger apoptosis in cancer cells via mitochondrial membrane disruption. In addition, certain HDPs are anti-angiogenic which may inhibit cancer progression. This paper reviews oncolytic HDP studies in order to address the suitability of selected HDPs as oncolytic therapies.

## 1. Introduction

The human immune system mainly recognizes and eliminates malignant cells via receptor-mediated mechanisms. The immune response can play a significant role in controlling malignant growth under natural conditions or in response to therapeutic manipulation, but malignant cells may evade immune surveillance [1,2].

Conventional cytotoxic therapies, such as radiation and chemotherapy, are the methods of choice for cancer management. Radiation therapy is relatively precise and used to achieve local control, whereas chemotherapy exerts a systemic effect and is used as a broad array of cancer treatment. However, both therapies have low therapeutic indices and are often highly toxic with a broad spectrum of severe side effects. Numerous chemotherapeutic drugs have been developed to treat cancers, including DNA-alkylating agents, antimetabolites, and hormone agonists/antagonists. Although these drugs have been successfully used for the treatment of metastatic cancers, severe side-effects and dose limitations are prevalent. This is because they are nonspecific and target any rapidly dividing cell without discriminating between healthy and malignant cells [3].

Moreover, cancer cells develop resistance to the available chemotherapeutic that is mediated by the overexpression of multidrug-resistance proteins that pump the drugs out of cells and thus render the drugs ineffective [4]. In addition, many neoplasms eventually become resistant to conventional chemotherapy due to selection for multidrug-resistant variants [5]. The limitations associated with existing chemotherapeutic drugs have stimulated the search for new oncolytic agents with new modes of action.

A promising family includes host defence peptides (HDPs) that recently have received attention as alternative chemotherapeutic agents that overcome the limits of current drugs. These peptides have several advantages over currently used oncolytic therapeutics, such as selective cytotoxicity for cancer cells, ability to bypass the multidrug-resistance mechanism, and additive effects in combination therapy [6].

### 1.1. Host Defense Peptides

HDPs are ubiquitous in nature. They have been found in animals as diverse as insects and human. Recent developments highlight the importance of vertebrate epithelial cells as sites of HDPs production and suggest a critical role for this system in human health and disease. Gene-encoded HDPs are found widely in the animal kingdom and salient examples include the cecropins from insects, magainins, temporins and dermaseptins from amphibians, and defensins from mammals [7–13]. These molecules tend to exhibit intrinsic specificity for microbial invaders and are relatively much less toxic for the metazoan host’s cells. However, some are also active on eukaryotic cells. This specificity endows the animal with an “innate” immunity, in contrast to the better studied acquired immunity conferred by the clonal expansion of B and T cells. The possible importance of this system as a check on infection is evident when one considers that most bacteria have generation times of 20–30 min whereas the mounting of a specific immune response, dependent on the growth of mammalian cells, may take days or weeks.

HDPs, capable of destroying prokaryotic and eukaryotic cell membrane attract peoples’ attention as they have different mechanism than the currently available drugs, which makes them candidates for novel therapeutic approaches.

### 1.2. Structure and Mechanism of Action

More than 1000 native HDPs have been isolated so far and they do not share a specific molecular composition, size, or a conformational structure. Their size vary between ~12–50 amino acids, some are linear and adopt α-helical or β-sheet structures in the presence of membranes, while others have specific folds due to the presence of disulfide bonds (e.g., defensins) [14–17]. Nevertheless, despite their structural diversity they have hallmark physical features of a high net positive charge and a threshold of hydrophobicity that allows them to adopt an amphipathic structure and to interact with the membrane of the microbes. HDPs are typically surface and membrane active agents that kill various cells by binding and disrupting the structural integrity of the cell membrane and as a result the homeostatic mechanisms of the target cell [9,18,19].

The positive charges serve to attract the antibiotic to the surface of the target microbe. The remarkable selectivity of these peptides is thought to be due to the relative abundance of negative charges in target cell membranes compared to those of the metazoan host. These membrane charges are due primarily to anionic phospholipids [18,19]. Such charges act as the “receptors” for the highly cationic HDOs. The amphipathic nature of the antibiotics is also a critical feature and allows them to insert into the membrane and permeate it via the formation of pores, holes, or ion channels [20]. Membrane permeation can take place via different mechanisms such as the “barrel stave” or the “carpet” to form organized channels or intermediate “toroidal” pores, respectively before membrane disintegration. Nevertheless, the exact molecular mechanism depends upon the specific molecule used [6,21].

The presence of cholesterol in host cell membranes may serve to protect them from the disruptive effects of the HDPs. Since microbes lack cholesterol in their membranes this phenomenon probably further increases the selectivity of these HDPs. In many instances cells with a higher transmembrane potential are more susceptible to the killing effects of HDPs. Such a potential, being negative on the inside of the cell, may provide an electromotive force that aids in driving the integration of the positively charged HDP into the target membrane [18]. There is an ample of evidence that the activity of most HDPs in not receptor mediated. For example, the fact that synthetic HDPs consisting of all d amino acids [21–23], both d- and l- amino acids or scrambled sequences [24,25] are as biologically active as their l amino acid containing counterparts. This observation underscores the uniqueness of the mechanism of action of HDPs as compared to most pharmaceuticals, which are direct inhibitors of enzymes with chiral active sites.

The oncolytic mechanisms of HDPs include: (i) triggering of necrosis via the cell membrane lytic effect [26–29]; (ii) trigger apoptosis via mitochondrial membrane disruption [26,27]; and (iii) non-membranolytic modes of action. Similar to prokaryotic cells, many cancer cells have a higher content of anionic phospholipids on their outer leaflet relative to normal eukaryotic cells. It has been demonstrated that HDPs induce cell death of transformed cells, but are much less cytotoxic to non-transformed cells [7,9,28–38].

These results suggest that HDPs may have future value as oncolytic drugs either alone or in combination with conventional therapies. The mechanisms of action of HDPs against prokaryotic cells have been widely investigated, while much less is known regarding the interactions with and effects on eukaryotic cells [14,19,39–41].

Pro-apoptotic effects induced by HDPs that are conjugated to a functional domain, which allow receptor-mediated or receptor-independent internalization in eukaryotic cells, have been reported. After internalization these HDP conjugates caused mitochondrial membrane disruption resulting in cytochrome c release and induction of apoptosis [27,42]. Other types of HDPs translocate spontaneously into the cell cytoplasm where they depolarize the inner mitochondrial membrane. Whether the mitochondria in intact cells are disrupted as a result of a direct effect of the HDPs is unknown [43,44].

**Figure 1:**
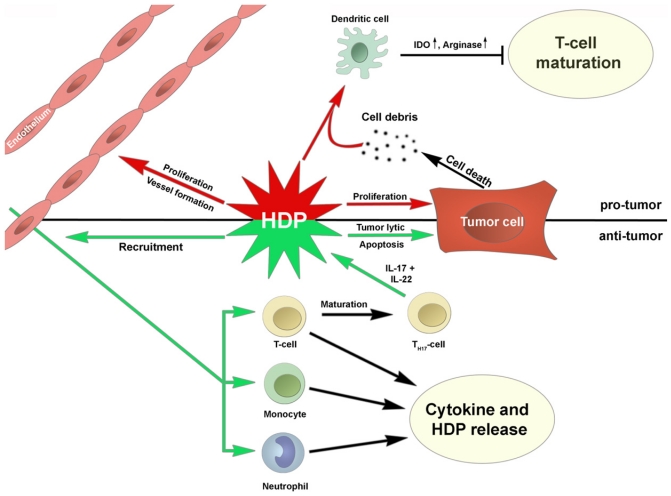
Potential functions of host defense peptides (HDPs) in cancer.

Exploiting the oncolytic properties of HDPs may embody a new paradigm for therapy of neoplasms. Despite the evidence that HDPs can play a significant role in controlling neoplastic growth, the cascade of molecular events leading to oncolytic activity remains to be fully elucidated (Figure 1). Nevertheless, some recent advances might drastically change the way of designing the next generation of cancer vaccines, hopefully improving the effectiveness of this therapeutic approach.

## 2. α-Helical Oncolytic Peptides

### 2.1. BMAP-27 & BMAP-28

There is evidence that these bovine cathelicidin-derived HDPs are cytotoxic and cause apoptosis of both freshly isolated leukaemia cells and certain human leukaemia cell lines [43,45,46]. Unfortunately, these HDPs have a narrow therapeutic dose range as low doses are only active against proliferating leukaemia cells and not dormant or slowly multiplying leukaemia cells and high doses cause haemolysis of benign red and white blood cells [45,46].

### 2.2. Cecropin A and B

Cecropin A and B belong to the Cecropin-family of antimicrobial peptides which were first isolated from the hemolymph of the giant silk moth Hyalophora cecropia [8,14,47]. Cecropins have the ability to form amphipathic alpha-helices which allow them to target nonpolar lipid cell membranes. Upon membrane targeting, they form ion-permeable channels subsequently resulting in cell depolarization, irreversible cytolysis and finally death [48,49]. Ceropin B has been shown to induce pore formation in cell walls [50,51]. Although some researchers have suggested that cecropins exert cytolytic activity against cancer cells through ion-permeable channel formation in the cell membrane, the precise mechanism of the cancer cell-killing action of the peptides remains to be deciphered. Cecropins kill neoplastic cells at concentrations lower than those required to lyse normal cells such as erythrocytes, fibroblasts and peripheral blood lymphocytes [32,52,53].

Studies have demonstrated specific oncolytic activity of both Cecropin A and B against mammalian leukemia, lymphoma and colon carcinoma cell lines [32,53,54] as well as small cell lung cancer [55] and gastric cancer cells [56]. *In vivo*, Cecropin B improves survival of mice bearing ascitic colon adenocarcinomas [53]. Transfection of human bladder cancer cells with Cecropin genes reduces their malignancy in nude mouse models [57]. Cecropin A and B have demonstrated significant inhibition of maliganant cell proliferation against bladder cancer cell lines by direct cancer cell lysis, probably by target cell membrane disruption [58]. While Cecropin A was able to induce apotosis in leukemia cells [59] and HL-60 a promyelocytic cell line [59], Cecropin B has been shown to have oncolytic activity *in vitro versus* multidrug-resistant human breast and ovarian cancer cell lines and *in vivo versus* mice with ascitic colon adenocarcinoma cells [53]. Polychemotherapy, of established agents 5-fluorouracil and cytarabine with cecropin A has a synergistic oncolytic effect on leukemia cells at certain doses [52].

### 2.3. LL-37/hCAP-18

Cathelicidin (LL37) consisting of the linear C terminus of the human CAP-18 molecule, has an alpha-helical structure, and interacts with the formyl peptide receptor-like 1 (FPRL1) G protein coupled receptor rather than with a chemokine receptor [60,61]. Cathelicidins are a family of antimicrobial proteins found in most mammalian species. They consist of a highly conserved *N*-terminal domain, cathelin, and a variable *C*-terminal peptide, which is proteolytically released upon demand. The single human member of this family, 18 kDa human cationic antimicrobial protein, hCAP18, is mainly produced by leucocytes and epithelial and mucosal cells. hCAP18 is the pro-protein and LL-37 is the mature processed form [62]. A 27-mer peptide of the *C*-terminal domain (hCAP18 (109–135)) of hCAP-18 which corresponds to amino acid residues 6–32 of LL-37, was recently found to induce apoptosis in a human oral squamous carcinoma cell line via a mechanism involving mitochondrial depolarisation without any detectable activation of caspase-3 [63].

It has been proposed that the the oncolytic activity of LL-37/hCAP-18 is due to LL-37(17–29). This is a 13 amino acid residue fragment of the *COOH*-terminal region corresponding to amino acid residues 17–29 [64]. This *C*-terminal domain of hCAP18, hCAP18109–135, induces apoptosis in both drug-sensitive and drug-resistant variants of oral squamous cell carcinoma SAS-H1 cells. In the same study, hCAP18109–135 had no cytotoxic effects on the HaCaT human keratinocyte cell line or on human gingival fibroblasts [63].

Unfortunately, LL-37 and its peptide fragments appear to be cytotoxic to the vascular system, being cytotoxic to both human peripheral blood leucocytes and untransformed endothelial cells with resulting haemolysis [65,66].

### 2.4. Magainins

Magainin II belongs to a family of antimicrobial peptides and was originally isolated from the skin of the African clawed frog, Xenopus laevis [67]. These peptides are important components in the innate host defense response in a wide range of organisms, from bacteria to humans [68]. Magainin II has an amphipatic α-helical structure that enables it to target both negatively-charged and nonpolar lipid cell membranes via the formation of toroidal pores, leading to depolarization, irreversible cytolysis, and finally to cell death [9,69].

Studies have demonstrated a significant cytotoxic effect of magainin II against a wide range of cancer cell lines including melanoma, bladder, breast and lung cancers as well as lymphomas and leukemias [7,35,70–72]. *In vivo*, magainins have been shown to improve survival of animals with ascites-producing neoplasms [7]. Furthermore, in a subcutaneous xenograft model of malignant melanoma growth in nude mice, local treatment of magainin II completely ablated the neoplasm [72]. More importantly, the main advantage of magainins is their selectivity for neoplastic *versus* normal cells. Magainin II peptide exerts cytotoxic and antiproliferative efficacy by membrane disintegration in bladder cancer cells but has no effect on normal murine or human fibroblasts [70].

Magainins kill neoplastic cells at concentrations lower than that required to lyse normal cells such as peripheral blood lymphocytes, erythrocytes, and normal murine or human fibroblasts [9,69,70]. Additionally, magainins are highly resistant to serum proteolysis [21]. Although some researchers have suggested that these peptides exert cytolytic activity against cancer cells through ion-permeable pore formation in the cell membrane, the precise mechanism of the cancer cell-killing action of the peptides remains to be deciphered. Magainins appear to permeabilise cell membranes differently; by a toroidal mechanism in bacterial cells and by the carpet mechanism against mammalian cells [73].

The selective oncolytic activity of magainin II has not yet been completely explained. Physicochemical attributes of cell membranes such as lipoprotein content and fluidity may explain the differences of the magainin effect. It has been suggested that the relatively large amounts of anionic substances, such as phosphatidyl serine in tumour cells, may particularly attract cationic magainins [7,10,11,18,74,75].

The potential of magainin II as an oncolytic drug is enhanced by the fact that its unique mechanism of cell killing is unaffected by the multidrug resistance (MDR) phenotype in human melanoma and small-cell lung carcinoma [35,76]. Significant oncolytic activity of the structurally and functionally related antimicrobial peptide Magainin II has been demonstrated against bladder cancer cell lines *in vivo* [70]. In clinical practice, the continual emergence of drug resistance hinders the activity of many standard oncolytic agents. MDR, such as the overexpression of a membrane-bound efflux pump, P-glycoprotein [77], renders drugs such as Adriamycin and the vinca alkaloids useless. These drugs enter cells by passive diffusion but are actively pumped out of the cell by P-glycoprotein. Magainin II, in contrast, has been found to have similar oncolytic activity against chemonaive cells as well as MDR cell lines [35,76,77]

### 2.5. Mellitin

Melittin is a HDP consisting of 26 amino acids and is the principal active component of bee venom. It is a powerful stimulator of phospholipase A2 and has been shown to specifically counterselect for cells in culture that express high levels of the ras oncogene [78]. However, due to its high toxicity against all types of cells it has not been investigated intensively as a potential oncolytic peptide.

## 3. B-Sheet Oncolytic Peptides

### 3.1. Defensins

The defensins can be classified into two subfamilies based on their tertiary structure. They exhibit considerable variation in their amino acid sequences, perhaps based on selective pressures to enable them to contend with a wide variety of microbial agents. The human defensins, although small (3.5–4 kDa), have three intramolecular cysteine bonds linking cysteines 1–6, 2–4, and 3–5, whereas the β defensins (4–6 kDa) have bonds between cysteines 1–5, 2–4, and 3–6. Consequently, these small molecules have an intricate tertiary structure with a core of three anti-parallel β-sheet components resembling chemokines [7,79,80].

Similar cysteine-rich HDPs, also called defensins, are found in plants, fungi and invertebrates and share many structural features and activities with human α and β-defensins [81]. Humans produce six different α-defensins, including 4 peptides (HNP-1 to HNP-4) in neutrophils and 2 peptides (HD5 and HD6) in Paneth cells of the small bowel. β-defensins, which are phylogenically older, more basic and slightly longer (36 or more amino-acid residues), form a rapidly growing family. The first human β-defensin hBD1 was identified in 1995. Five other hBD peptides have been partly characterized in the following years and a recent analysis of the human genome identified almost 40 potential coding regions for β-defensins [82].

α-defensin and β-defensin families do not share DNA sequence similarity or disulfide topology but possess highly similar tertiary structures with comparable antimicrobial activity [81]. HNP1 could also inhibit angiogenesis by affecting include lung cancers, renal cell carcinomas, squamous cell carcinomas of the tongue, bladder carcinomas and endothelial cell proliferation, adhesion and migration [83–88]. However, at lower doses, HNP1–3 can also increase the proliferation, motility and invasiveness of cancer cell lines [87,89]. HNP-1 is oncolytic against several cancer cell lines, including Raji human B-lymphoma cells, human oral squamous carcinoma cells, and MOT mouseteratocarcinoma cells [10,11,79,90]. In addition, concentrations of HNP1–3 higher than 25 μg/mL exert cytotoxic effects on both eukaryotic cells and cancer cell lines [10,79,88,90], and the presence of α-defensins in tumor cells explored by immunohistochemistry is sometimes associated with morphological signs of necrosis in surrounding cells [88]. Rabbit macrophage-associated defensins that are homologues of HNP-1 and -2 are also able to lyse murine cancer cells [91].

Cancer cell killing by HNP-1, -2, and -3 involves a membrane binding event, most likely mediated by electrostatic interactions, followed by rapid collapse of the membrane potential and loss of membrane integrity [10,11]. Membrane permeabilisation by HNPs has been attributed to the channel-forming ability of these peptides because HNP-1 has been shown to form voltage-dependent, ion-permeable channels in planar phospholipid bilayer membranes [92].

HNP-mediated cytotoxicity may also involve DNA damage since single strand DNA breaks were detected in HNP-treated target cells, although nucleosome-sized fragments of DNA that are characteristic of apoptosis were not observed [93]. In addition to their cytotoxic activity, HNP-1 and -3 may be able to interfere with neovascularisation during cancer development because these α-defensins inhibit the α5β1 integrin-dependent migration and adhesion of endothelial cells to fibronectin in response to vascular endothelial growth factor (VEGF) [84]. In addition, HNP-1 and -3 inhibited VEGF-induced proliferation of endothelial cells via the induction of apoptosis. Unfortunately, the clinical utility of human α-defensins is limited by the fact that HNPs are not cancer-selective, causing the lysis of normal human leukocytes, epithelial cells, and fibroblasts [10,94]. Furthermore, intratumoral overexpression of HNP-1 mediates inhibition and partially eradication of breast and colon carcinoma by modulation of anti-tumor immunity, induction of apoptosis or inhibition of angiogenesis [95]. In addition, serum strongly inhibits HNP-mediated cytotoxicity, which poses an obstacle to the systemic administration of these human α-defensins [11].

Defensins are also potent immunological adjuvants and could be used to overcome the ability of malignant cells to evade immunosurveillance [96,97]. For example, defensins recruit dendritic cells in organotypic cultures of HPV-transformed keratinocytes maintained *in vitro* or grafted *in vivo*, suggesting that these molecules may restore some immune functions altered during cervical carcinogenesis [98]. Therefore, defensins could be useful in the formulation of therapeutic oncolytic vaccines via chemotactic recruitment of dendritic cell precursors that may promote neovascularization, in part by undergoing endothelial-like trans-differentiation [99]. α- and β-defensins could provide an endogenous balance in angiogenesis regulation, depending on the extent to which recruitment of dendritic cells or neutrophils predominates. The anti-angiogenic effects of α-defensins could contribute to their antitumour effect [100].

*In vitro*, α-defensins are atypical ligand of α5β1-integrin that specifically inhibits α5β1-integrin-dependent migration of endothelial cells to fibronectin. They also attenuate the VEGF-stimulated increase in endothelial permeability and block endothelial cell proliferation and capillary sprout formation in 3-dimensional fibrin-matrices [85].

### 3.2. Lactoferricin

Bovine lactoferricin is a peptide fragment produced by acid-pepsin hydrolysis of lactoferrin obtained from cow’s milk [101]. Lactoferricin, which consists of amino acid residues 17 to 41 proximal to the NH_2_ terminus of bovine lactoferrin. LfcinB also possesses potent *in vivo* activity against cancer cells [102–104]. Lactoferricin B binds to cancer cell membranes, causing membrane integrity to be lost due to the formation of transmembrane pores that allow the HDP to enter the cytoplasmic compartment of the malignant cell and co-localize with negatively-charged mitochondria [105,106]. Although mouse fibrosarcoma cells and human neuroblastoma cells exposed to lactoferricin B die primarily via necrosis caused by a cell membrane lytic effect [105], lactoferricin B kills human leukemia and breast carcinoma cells by a process that involves the sequential generation of reactive oxygen species, loss of mitochondrial transmembrane potential, and activation of the caspase cascade culminating in cell death by apoptosis [104,106]. Whether lactoferricin B-treated cancer cells die by necrosis or apoptosis may ultimately be determined by the degree of irreparable lactoferricin B-mediated damage to the cytoplasmic membrane relative to mitochondrial membrane damage caused by internalized lactoferricin B. Although the cytotoxic activity of lactoferricin B is reduced in the presence of high concentrations of serum [104], systemic or intratumoural administration of Lactoferricin B is nonetheless able to inhibit the *in vivo* growth and/or metastasis of several different cancer types in mice [33,104,105].

Lactoferricin B has been shown to suppress both basic fibroblast growth factor (bFGF)- and VEGF-driven proliferation and migration of human endothelial cells *in vitro*, as well as interfering with bFGF- and VEGF-induced angiogenesis in subcutaneous Matrigel plugs implanted in mice, by competing with bFGF and VEGF for growth factor receptor-associated heparan sulfate proteoglycans on the endothelial cell surface [106].

Administration of lactoferricin B to mice 1 day after intravenous cancer inoculation results in a significant inhibition of lung and liver metastasis by L5178Y-ML25 murine lymphoma cells as well as lung metastasis by B16-BL6 murine melanoma cells [104]. The same study showed an inhibitory effect by lactoferricin B on both cancer-induced angiogenesis and cancer growth in mice inoculated intradermally with B16-BL6 melanoma cells. In addition, intratumoural injections of lactoferricin B slow the growth of murine Meth A fibrosarcoma cells grown as subcutaneous cancers in mice [104]. Furthermore, oral administration of lactoferricin B to rats that had been injected previously with azoxymethane to promote colon carcinogenesis results in an impressive 83% reduction in the incidence of colon adenocarcinomas [103]. A similar effect was achieved by oral administration of intact bovine lactoferrin, which raises the intriguing possibility that lactoferricin B derived from dietary bovine lactoferrin may protect against colon carcinogenesis. Interestingly, consumption of milk and milk products has been linked recently to a reduced risk of colorectal cancer in humans [102] as well as reduced tumor growth in mice treated with 1,2-dimethylhydrazine to induce colon cancer [107].

Not much is known about the mechanism by which lactoferricin B exerts its oncolytic activity, although the available evidence favors a direct inhibitory effect of lactoferricin B on cancer cell growth and metastasis or induces apoptosis in Jurkat T-leukemia cell lines by affecting the ceramide pathway [108,109]. Studies indicate that *in vitro* exposure to lactoferricin B causes several different human leukemia and carcinoma cell lines to lose membrane integrity and eventually lyse [33,104–110]. In addition, lactoferricin B is a potent inducer of apoptosis in cultures of THP-1 human monocytic leukemia cells [111]. However, the anti tumor activity of lactoferricin B can be inhibited if the plasmamembran contains haparan sulfate on its surface [112].

### 3.3. Tachyplesin

Tachyplesin is a small HDP composed of 17 amino acids and isolated from the horseshoe crab. It has an amphipathic structure conferred by two antiparallel β-sheets held rigidly in place by two disulfide bonds [113]. A multistep model has been proposed to account for the oncolytic activity of tachyplesin [114]. First, tachyplesin binds to hyaluronan (or closely related glycosaminoglycans) on the surfaces of the target cells. Second, this bound tachyplesin interacts with C1q in the blood. Third, the tachyplesin activates the classic complement pathway. Finally, the formation of the MAC disrupts the integrity of the plasma membrane and results in the death of the target cells. Tachyplesin binds to both hyaluronan on the cell surface and C1q in the serum and activates the classic complement cascade, which damages the integrity of the membranes of the tumor cells resulting in their death [114]. The interaction between tachyplesin and hyaluronan could account for the relative specificity of oncolytic activity of tachyplesin. Indeed, several studies have shown that many malignant cells (bladder, prostate, lung, colon, and breast) express high levels of hyaluronan whereas normal tissues express much less [115–117]. In addition, studies have shown that endothelial cells involved in neovascularization express high levels of hyaluronan relative to those from established blood vessels [118]. The fact that hyaluronan is preferentially expressed on the surfaces of malignant and endothelial cells involved in cancer vascularisation could account for the observations that tachyplesin inhibits the growth of these cells relative to benign cell lines that express less hyaluronan on their surfaces. Tachyplesin has been shown to inhibit malignant growth in the presence of normal serum even against cells that overexpress the multiple-drug resistant gene [114]. A synthetic version of tachyplesin conjugated to the integrin homing domain RGD blocked the growth of tumor cells both *in vitro* and *in vivo* [26].

RGD-tachyplesin has been shown to inhibit the proliferation of both cultured malignant and endothelial cells and reduced the colony formation of TSU cancer cells. *In vivo*, it has been reported to inhibit the growth of neoplasms via induction of apoptosis in both malignant and endothelial cells evidenced by activation of several caspases in both the mitochondrial and Fas-dependent pathways [114].

## 4. Hybrid and Synthetic HDPs

### 4.1. Buforin II and Buforin IIb

Buforin II and IIb are HDPs with a helix-hinge-helix structure derived from histone H2A. They have a unique mechanism of action as they rapidly crosses bacterial membranes without lysing cells and kill by interacting with intracellular macromolecules [119]. Buforin IIb, is a hybrid peptide with stronger cytolytic activity against cancer cells than buforin II. Buforin IIb has been shown to display strong, selective oncolytic activity against a broad spectrum of cancer cells. Buforin IIb has selective oncolytic cell membrane penetration through interaction with gangliosides and then induces apoptosis in cancer cells by a mitochondria-dependent pathway, as confirmed by caspase-9 activation and cytochrome c release to cytosol as well as by DNA laddering and annexin V staining [120].

### 4.2. Hunter-Killer Peptides

Some novel peptides, called hunter-killer peptides (HKPs), have been designed to target and then kill malignant blood vessels without harming the normal vasculature. They comprise two functional domains, one a malignant blood vessel “homing” motif and the other a sequence inducing apoptosis. These HKPs home in on the malignant vascular system and therefore offer a less toxic alternative to current cancer therapies into native molecules, use of peptomimetics and peptide engineering are being developed. Research has also been focused on HKP delivery methods to further enhance the potential of these oncolytic HKPs. Furthermore, combination therapies are being developed that take advantage of multifunctional HKPs or place different active HKPs together (for example to induce apoptosis, necrosis, or immunostimulation), which together represent a novel therapeutic approach for oncolytic therapy and, it is anticipated, will develop into an inventive approach to tackle the problem of drug resistance [121].

Nude mice carrying MDA-MB-435 derived human breast carcinoma xenografts were treated with HKP-1 and HKP-2. Malignancies treated by HKP-1 reduced the size of neoplasms were on average 90% smaller than the size of tumors in the control groups and some of the HKP-1 treated nude mice outlived the controls by several months, indicating that both primary neoplasm growth and metastasis were inhibited by HKP-1. Lung metastases treated by HKP-1 and HKP-2 were also reduced in number and size [27,121]. HKP-3 was evaluated for efficacy in a mouse model of prostate cancer and extended the lives of treated mice by up to 2 months longer (nearly one-fifth of their normal life span) than that of control treated mice [122].

### 4.3. Polybia-MPI

Polybia-MPI (IDWKKLLDAAKQIL-NH2, 1.65 kDa) is a HDP from the venom of the social wasp *Polybia paulista*. It has been shown to selectively inhibit the proliferation of prostate and bladder cancer cells, but has lower cytotoxicity to normal murine fibroblasts or rat erythrocytes [2,123]. Polybia-MPI exerts cytotoxic and antiproliferative efficacy either by membrane disruption or by pore formation [2]. Once the bi-layer is disrupted, the transmembrane electrochemical potential collapses and cell death occurs in minutes [124]. So the surface charge of cell is important for peptide association to the membrane. Malignant cells are negatively charged, so they are prone to bind cationic amphipathic antimicrobial peptides. Additionally they have high transmembrane potentials, which can be disturbed by destabilization of the membrane, resulting in leaking of electrolytes and consequently, leading to cell death. This may explain how polybia-MPI kills the malignant cells.

Three analogs of polybia-MPI in which Leu7, Ala8 or Asp9 replaced by l-Pro have been designed and synthesised and l-Pro substitution of Leu7 or Asp9 significantly reduced the content of alpha-helix conformation, and l-Pro substitution of Ala8 can disrupt the alpha-helix conformation thoroughly. The l-Pro substitution induces a significant reduction of oncolytic activity, indicating that the alpha-helix conformation of polybia-MPI is important for its oncolytic activity [2]. Polybia-MPI demonstrates *in vitro* activity against multi-drug resistant leukemic cell and was able to reduce tumor growth of S180 mouse sarcoma after i.p. injection of 10 mg/kg of this peptide [125,126].

### 4.4. Epinecidin-1

Epinecidin-1 is a synthetic 21-mer antimicrobial peptide originally identified from Epinephelus coioides. Epinecidin-1 contains a prepropeptide of 67 amino acids, and the epinecidin-1 gene is composed of three epinecidin-1 peptides from the fish genome [127,128]. Epinecidin-1 has been demonstrated to have *in vitro* oncolytic and antiproliferative actions against the several cancer cell lines including, A549 (human lung carcinoma cell), HeLa (human cervix adenocarcinoma cell), HepG2 (human hepatocellular carcinoma cell), HT1080 (human fibrosarcoma cell), U937 (human histiocytic lymphoma), NIH3T3 (mouse fibroblast cell), RAW264.7 (mouse macrophage from a tumor induced by the Abelson murine leukemia virus), WS-1 (human kidney cell), AML-12 (murine hepatocyte cell) and HA59T/VGH (human hepatic tumor-derived cell). Epinecidin-1 has been shown to increase the cytotoxicity of these cancer cell lines in dose- and time-dependent manners, indicating that cell death occurred by membrane disruption [129,130].

### 4.5. d,l-Amino Acids Host-Defence-like Peptides

A novel family of antimicrobial peptides was developed, which are composed of Lys and Leu both in the forms of d- and l-isomers (diastereomers) [25,131]. It has been found that some of these peptides are highly selective towards cancer cell lines compared with normal cells. Most important, they could target and arrest the growth of aggressive and hormone-resistant, primary human prostate and breast tumors and prevent their experimental and spontaneous metastases, respectively, when intratumor [28] or systemically [29] inoculated to immune-deficient mice. These effects were correlated with increased necrosis of the tumor cells and a significant decrease in the overall tumor microvessel density, as well as newly formed capillary tubes and prostate-specific antigen secretion (in prostate tumors). Mode of action studies revealed that the exclusive selectivity of the peptides towards cancer derives from their specific binding to surface phosphatidylserine and the killing of the cancer cells via cytoplasmic membrane depolarization. Interestingly, the acidic environment created by solid tumors could be used as a trigger to activate another family of diastereomeric peptides, by making them cationic only at low pH levels. This could be achieved by substituting Lys with His in the diastereomeric family of peptides. His is protonated only at acidic pH environments. Such peptides had also reduced systemic toxicity [132]. In addition, [d]-K_3_H_3_L_9_ has been demonstrated to induce full tumor remission, partial remission or growth reduction in cases of aggressive liposarcomas, synovial sarcoma cells or murine fibrosarcoma (BFS-1), when intratumor inoculated to immune-deficient or C57BL/6 mice respectively [133,134].

d-peptide A, B, C and D are four enantiomeric 9-mer peptides designed and synthesized on the basis of 43-mer beetle defensins. These cationic and amphipathic d-9-mer peptides (net charges of +3 to +5) maintain bacterial membrane disruptive activity similar to the original peptides and have been shown to have selective oncolytic activity, against several different cancer cell lines and similar antimicrobial mechanisms similar to the original defensins (membrane disruption), but no cytotoxicity against mammalian erythrocytes, leucocytes, macrophages and fibroblasts [18,135–138].

Overall, diastereomeric peptides have important advantages over native antibacterial and conventional peptides therapeutics. These include: (1) Their simple compositions; (2) Their ability to preserve activity in serum; (3) They lack the diverse pathological and pharmacological effects attributed with peptide therapy; (4) They have very low or no antigenicity; (5) Their lytic mode of action makes it difficult for the target to develop resistance.

## 5. Discussion

HDP research is becoming an area of great importance and understanding the role of HDPs in humans is in its infancy. The number and types of these HDPs, their tissue specific expression and genetic regulation all remain to be addressed. Understanding these aspects may ultimately allow manipulation of this arm of the immune system for the benefit of human health, analogous to stimulation of the cellular and humoral immune system by vaccination. In particular, the part of the arrangement of the phospolipid bilayers of the cells in the biological action of these HDPs continues to be contentious. There are a number of actions in which cell membranes may be engaged in the functioning of these HDPs. HDPs may form specific holes/channels in the cell membranes, allowing seepage of intracellular ions and other cell contents [139–144]. On the other hand, HDPs may cause disarray of both cell and mitochondrial membranes through the “carpet” mechanism [145,146].

The emergence of resistance to standard chemotherapeutic agents has increased the urgency of exploring alternative means of combating neoplasia. HDPs, as oncolytics, have potentially advantageous attributes. HDPs are rapidly emerging as attractive candidates for oncolytic treatment. Although many HDPs are highly toxic against malignancies cancer *in vitro*, their potential for applications depends on additional features. As a result of their cationic and amphiphilic properties, the peptides may bind to host components which reduces their bioavailability and generates harmful side effects, such as lysis of red blood cells [147]. This amphipathic design, consisting of spatially separated hydrophobic and charged regions, permits intercalation of the HDPs peptide into cell membranes. Their unique mechanism of action and ability (in many cases) to kill neoplastic cells makes them attractive prototypes for oncolytic drug development. It has also been established that many also have a key modulatory role in the innate immune response and form an important link between the innate and adaptive immune responses [148]. Owing to their multiple functions, they are considered promising agents for new oncolytic therapeutic approaches. Many more HDPs remain to be discovered and this class of compounds may soon represent a new weapon against human cancers.

This manuscript underscores the broad oncolytic potential of HDPs and suggests a new strategy for treatment. Future basic and clinical research will tell if and when this new powerful “biological weapon” will become part of the health professionals’ armentarium. Further research of HDPs will improve our knowledge of their involvement in the recognition and lysis of neoplastic cells, which will support the development of new oncolytic therapeutic approaches. A short overview of studied HDPs and their potential therapeutic options is given in Table 1. HDP immunomodulating functions will become clearer with additional research on structure-function analyses to elucidate their mechanisms of action. Most important, future research must take advantage of and build on the diverse nature of HDPs and adhere to physiologically relevant conditions, ultimately validating, *in vivo*, their beneficial functions to treat malignancies.

## 6. Conclusion

It is clear that the enormous potential of HDPs to serve as oncolytic drugs has re-energized the scientific community in the search for better ways to combat malignancies. The effectiveness of any targeted therapeutic largely depends on whether the appropriate drug can be delivered to the desired location in sufficient quantities and in a timely fashion. Despite many technical hurdles, such as weak efficiency *in vivo* and adverse toxicity, HDPs are advantageous because they are small, specific to malignant cells, possess low immunogenicity and are relatively easy to modify and design. HDPs and their modified derivatives will advance to clinical trials. Research must also be focused on HDP delivery methods to further enhance their oncolytic potential. Furthermore, combination therapies are being developed that take advantage of multifunctional HDPs or place different active HDPs together, for example to induce necrosis, apoptosis or immunostimulation, which together represent a novel therapeutic approach for oncolytic therapy and, it is hoped, will develop into an innovative approach to address the problem of drug resistance. Today’s challenge is to design an oncolytic peptide with optimised properties. However, the exploitation of recent insights into cancer biology and its microenvironment combined with a multidisciplinary approach involving chemistry, biology, high-throughput instrumentation and formulation science will hopefully lead to the discovery of new, more-effective, tumor-targeting HDPs.

## Figures and Tables

**Table 1:** Evidence for oncolytic properties of HDPs.

Peptide	Cancer cell	Study	Reference
Polybia-MPI	Human bladder and prostate	*In vitro*, compared against murine fibroblasts	Wang *et al*. 2008
Tachyplesin	Prostate cancer cell line	*In vitro* and syngenic mouse model	Chen *et al*. 2001
Buforin IIb	62 different cancer cell lines	*In vivo* mouse xenograft model	
	Human breast cancer cell line	*In vitro*	Furlong *et al*. 2006
	Human neuroblastoma cell lines	*In vitro* and neuroblastoma mouse xenograft model	Eliassen *et al*. 2006
Bovine lactoferricin	Jurkat T-leukemia cells	*In vitro*	Mader *et al*. 2007
	adenocarcinoma	*In vivo* tumor induced F344 rats	Tsuda *et al*. 1998
	Human monocytic leukemic cells	*In vitro*	Yoo *et al*. 1997
Cecropin A and B	Bladder cancer cells	*In vitro*	Suttmann *et al*. 2008
Human alpha-defensin-1	Human lung adenocarcinoma	Mouse xenograft model	Xu *et al*. 2008
Human alpha-defensin-1 and lactoferrin	oral squamous cell carcinoma	*In vitro*	McKeown *et al*. 2006
Human beta-defensin-1	Prostate cancer cell lines	*In vitro*	Bullard *et al*. 2008
hCAP-18_(109–135)_	oral squamous cell carcinoma	*In vitro*	Okumura *et al*. 2004
Magainin II	Bladder cancer cell line hematopoietic tumor and solid tumor target cells	*In vitro*	Lehmann *et al*. 2006
		*In vitro*	Cruciani *et al*. 1991
Magainin II and derivatives	Several cell lines	*In vivo* ovarian cancer model	Baker *et al*. 1993
MSI-511 (Magainin derivative)	Several human melanoma cell lines	*In vitro*	Soballe *et al*. 1995
Magainin A and G	Small cell lung cancer cell lines	*In vitro*	Ohsaki *et al*. 1992
Sythetic 9-mer d-peptides	Mouse myeloma cell line	*In vitro*	Iwasaki *et al*. 2009
CA-ME (hybrid peptide)	Small cell lung cancer cell lines	*In vitro*	Shin *et al*. 1997
	Prostate carcinoma cell lines	Mouse xenograft model	Papo *et al*. 2004
Synthetic l/d-peptides	Primary human prostate and breast cancer cells	Prevents metastases in mouse xenograft model	Papo *et al*. 2006
	Mouse melanoma and lung cancer cell lines	Mouse xenograft model	Papo *et al*. 2003
	Human liposarcoma and synovial sarcoma cells	Mouse xenograft model	Steinstraeser *et al*. 2011
